# Predictors of Exercise Capacity in Patients with Hypertrophic Obstructive Cardiomyopathy

**DOI:** 10.3390/jcm7110447

**Published:** 2018-11-18

**Authors:** Joshua R. Smith, Jose R. Medina-Inojosa, Veronica Layrisse, Steve R. Ommen, Thomas P. Olson

**Affiliations:** 1 Division of Preventive Cardiology, Department of Cardiovascular Medicine, Mayo Clinic, Rochester, MN 55095, USA; medinaInojosa.jose@mayo.edu (J.R.M.-I.); veronicall@sanjuanbautista.edu (V.L.); ommen.steve@mayo.edu (S.R.O.); olsont.thomas2@mayo.edu or olson.thomas2@mayo.edu (T.P.O.); 2 San Juan Bautista School of Medicine, Caguas, PR 00727, USA

**Keywords:** hypertrophic cardiomyopathy, exercise testing, exercise capacity

## Abstract

Hypertrophic obstructive cardiomyopathy (HOCM) patients exhibit compromised peak exercise capacity (VO_2_peak). Importantly, severely reduced VO_2_peak is directly related to increased morbidity and mortality in these patients. Therefore, we sought to determine clinical predictors of VO_2_peak in HOCM patients. HOCM patients who performed symptom-limited cardiopulmonary exercise testing between 1995 and 2016 were included for analysis. Peak VO_2_ was reported as absolute peak VO_2_, indexed to body weight and analyzed as quartiles, with quartile 1 representing the lowest VO_2_peak. Step-wise regression models using demographic features and clinical and physiologic characteristics were created to determine predictors of HOCM patients with the lowest VO_2_peak. We included 1177 HOCM patients (age: 53 ± 14 years; BMI: 24 ± 12 kg/m^2^) with a VO_2_peak of 18.0 ± 5.6 mL/kg/min. Significant univariate predictors of the lowest VO_2_peak included age, female sex, New York Health Association (NYHA) class, BMI, left atrial volume index, E/e’, E/A, hemoglobin, N-terminal pro b-type natriuretic peptide (NT-proBNP), and a history of diabetes, hypertension, stroke, atrial fibrillation, or coronary artery disease. Independent predictors of the lowest VO_2_peak included age (OR, CI: 1.03, 1.02–1.06; *p* < 0.0001), women (4.66, 2.94–7.47; *p* = 0.001), a history of diabetes (2.05, 1.17–3.60; *p* = 0.01), BMI (0.94, 0.92–0.96; *p* < 0.0001), left atrial volume index (1.07, 1.05–1.21; *p* = 0.04), E/e’ (1.05, 1.01–1.08; *p* = 0.004), hemoglobin (0.76, 0.65–0.88; *p* = 0.0004), and NT-proBNP (1.72, 1.42–2.11; *p* < 0.0001). These findings demonstrate that demographic factors (i.e., age and sex), comorbidities (e.g., diabetes and obesity), echocardiography indices, and biomarkers (e.g., hemoglobin and NT-proBNP) are predictive of severely compromised VO_2_peak in HOCM patients.

## 1. Introduction

Hypertrophic cardiomyopathy (HCM) is a commonly inherited heart disease affecting 1 in 500 individuals [1], with ~70% of these patients developing the obstructive phenotype, hypertrophic obstructive cardiomyopathy (HOCM) [2]. Patients with HOCM have a greater risk of mortality and greater severity of heart failure than HCM patients without obstruction [3]. HCM patients with and without obstruction generally present with exertional dyspnea, fatigue, and reduced peak functional capacity [1]. Factors contributing to the diminished peak functional capacity include impaired stroke volume response, left ventricular systolic and diastolic dysfunction, chronotropic incompetence, and peripheral muscle changes [1,4,5,6].

Cardiopulmonary exercise testing (CPET) is recommended for HCM patients to determine the severity of exercise intolerance, and the responsible mechanisms [7,8]. CPET also has important, clinically useful prognostic utility in HCM [9,10,11,12,13]. Specifically, reduced peak oxygen consumption (VO_2_peak) in HCM patients is associated with greater mortality and more severe progression of heart failure [9,10,12,13]. Furthermore, obstruction is associated with a further reduction in VO_2_peak in HCM [9,10,14,15]. HCM patients also exhibit numerous cardiovascular risk factors, including diabetes, arterial hypertension, and coronary artery disease [9,10,11,16,17]. However, the contribution of these cardiovascular risk factors to the worsening of VO_2_peak in patients with HOCM is unknown. Therefore, in the present study we sought to determine the relationship(s) between demographic factors, comorbidities, and echocardiography indices with the compromised VO_2_peak in HOCM patients.

## 2. Experimental Section

*Study design:* This was a retrospective observational study of 1177 consecutive patients with HOCM tested in a single center between 1995 and 2016. All patients included in this analysis performed symptom-limited CPET testing and a comprehensive transthoracic echocardiogram within one week. Clinical CPET and echocardiographic data were obtained from an institutional database. A random sample in the electronic medical record was reviewed independently and in duplicate by two investigators (J.M.I. and V.L.) to validate the research strategy. This study was approved by the Mayo Clinic Institutional Review Board, and all patients agreed to the use of their medical records for research.

*Clinical and echocardiographic assessment:* HOCM diagnosis was based on clinical and echocardiographic evaluation by a cardiologist, assessing evidence of myocardial hypertrophy in the absence of cardiac or systemic disease associated with hypertrophy. Systemic hypertension did not preclude HOCM diagnosis if the myocardial hypertrophy was greater than clinically expected from the systemic hypertension. All resting transthoracic echocardiogram measurements were performed according to the guidelines of the American Society of Echocardiography. Of note, continuous wave Doppler and the modified Bernoulli equation (i.e., gradient = 4*v*^2^, where *v* = peak velocity) were used to determine left ventricular outflow tract gradient. Echocardiographic evidence of HOCM included left ventricular hypertrophy (i.e., septal thickness ≥15 mm) and left ventricular outflow gradient of ≥30 mmHg [8]. If obstruction was not present at rest, provocation via Valsalva maneuver, amyl nitrite, and/or exercise was performed [8].

*Cardiopulmonary exercise testing:* Clinically indicated CPET was performed by exercise physiologists under the supervision of a cardiologist. Patients completed an institutionally designed incremental exercise protocol [18,19] on a motor-driven treadmill (GE CASE, Milwaukee, WI, USA). Briefly, this protocol consisted of increasing two metabolic equivalents every two minutes until volitional fatigue [18,19]. To maximize generalizability and minimize potential for decompensation, cardiac medications were not withheld prior to CPET. Ventilatory and metabolic variables were measured during exercise (MGC Diagnostics, St. Paul, MN, USA). Peak VO_2_ was the highest 30 s averaged value, expressed as L/min and mL/kg/min. The percent (%) predicted peak VO_2_ was reported using the FRIEND equation [20], and a % predicted peak VO_2_ of <80% was defined as abnormal [13,15,21,22]. Abnormal VO_2_ slope change was determined by a plateau in the VO_2_ versus time relationship, assessed by visual inspection. For patients not prescribed beta blocker therapy, peak heart rate was predicted using 220 − age. For patients on beta blocker therapy, 119 + (0.5 × resting heart rate) − (0.5 × age) was used to determine predicted peak heart rate [23]. The O_2_ pulse (mL/beat) was determined by dividing VO_2_ by heart rate. The predicted peak O_2_ pulse was calculated by dividing the predicted VO_2_peak by the predicted peak heart rate [19]. The O_2_ pulse slope change was assessed visually as the relationship between O_2_ pulse versus time. An abnormal O_2_ pulse was defined as a plateau in O_2_ pulse during exercise. Forced expiratory volume in 1 s (FEV_1_) (reported as % predicted) and breathing reserve were determined according to ATS/ERS guidelines [24]. 

*Statistical analysis:* Statistical analyses were performed using JMP software version 13.0 (SAS Institute Inc., Cary, NC, USA). Variables are presented as mean ± standard deviation (SD) or absolute number of patients (% of total sample) and were compared using one-way analysis of variance, Pearson chi-square test, or Fisher’s exact test accordingly. Normality was assessed by visual assessment. Univariate and multivariate logistic regression models were created to test for clinical and echocardiographic predictors of low peak relative VO_2_. Based on previous studies [9,13,15,21], we anticipated that the majority of our sample would have an abnormal peak VO_2_. Therefore, we grouped our sample in evenly distributed quartiles according to peak VO_2_ using the lowest (i.e., quartile 1) as the referent group in a similar manner as Coats et al. [12]. Univariate modeling was performed adjusting for age, sex, clinical, and echocardiographic factors known to potentially affect peak VO_2_, and those that were significant were included in the final multivariate modeling. Odds ratio (OR) and 95% confidence intervals (CIs) are presented. Missing data were handled by omission from final models. Statistical significance was set at *p* < 0.05.

## 3. Results

The sample population included 676 men and 501 women, and the mean age was 53 ± 14 years. Demographics, clinical characteristics, and cardiovascular risk factors according to VO_2_ quartiles (quartile 1: ≤14.14; quartile 2: 14.15–17.70; quartile 3: 17.71–21.51; quartile 4: ≥21.52 mL/kg/min) are presented in Table 1. Significant differences across quartiles were present for age, height, weight, BMI, FEV_1_ (% predicted), hemoglobin, NT-proBNP, sex, New York Health Association (NYHA) class, dyslipidemia, smoking history, and beta blocker use. Resting echocardiographic data stratified by VO_2_peak quartile are presented in Table 2. Left ventricular ejection fraction, septum thickness, medial E/e’, and mitral E/A were significantly different across the quartiles. Table 3 shows the peak CPET data stratified by peak VO_2_. The average VO_2_peak for the entire sample population was 18.0 ± 5.6 mL/kg/min, with 1172 (99.6%) of the HOCM patients achieving ≤80% predicted VO_2_peak. At peak exercise, significant differences were present in treadmill time, VO_2_, respiratory exchange ratio, heart rate, breathing reserve, O_2_ pulse, % predicted O_2_ pulse, V_E_/VCO_2_, abnormal VO_2_ slope, and abnormal O_2_ pulse slope across quartiles.

**Figure 1:**
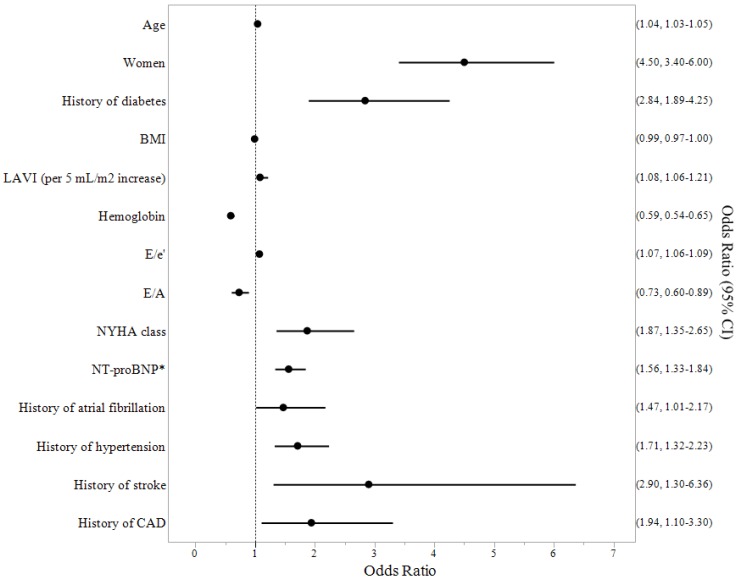
Univariate predictors of VO_2_peak in hypertrophic obstructive cardiomyopathy (HOCM) patients. Significant predictors of the lowest VO_2_peak were age, female sex, BMI, left atrial volume index (LAVI, per 5 mL/m^2^ increase), hemoglobin, E/e’, E/A, NYHA class, NT-proBNP, and histories of atrial fibrillation, hypertension, stroke, and coronary artery disease (CAD). * per each SD increase. Odds ratio and 95% confidence interval shown for each variable.

**Figure 2:**
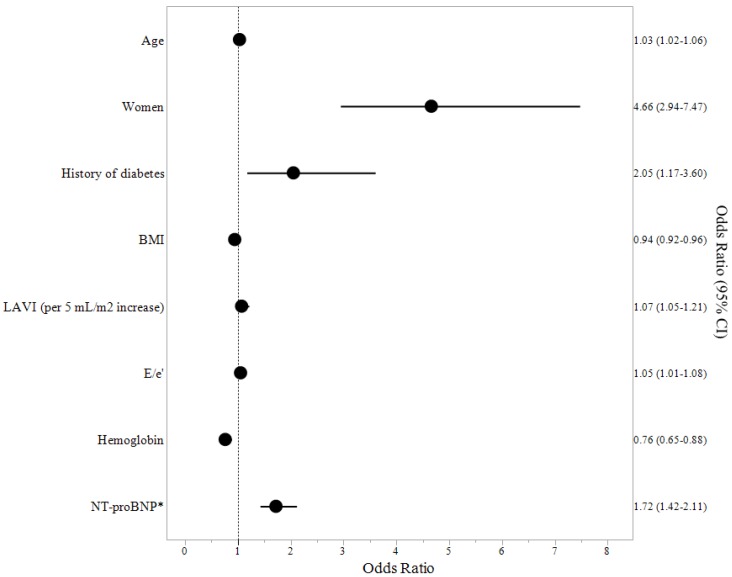
Multivariate predictors of VO_2_peak in HOCM patients. Significant independent predictors of the lowest VO_2_peak were age, female sex, a history of diabetes, BMI, left atrial volume index (LAVI, per 5 mL/m^2^ increase), E/e’, hemoglobin, and NT-proBNP. * per SD increase. Odds ratio and 95% confidence interval are shown for each variable.

Figure 1 shows the univariate predictors of VO_2_peak in HOCM patients. Age (*p* < 0.0001), female sex (*p* < 0.0001), NYHA class (*p* = 0.0003), BMI (*p* = 0.03), hemoglobin (<0.0001), NT-proBNP (*p* < 0.0001), left atrial volume index (*p* = 0.006), E/e’ (*p* < 0.0001), E/A (*p* < 0.0001), as well as a history of diabetes (*p* < 0.0001), atrial fibrillation (*p* = 0.04), hypertension (*p* < 0.0001), stroke (*p* = 0.01), and coronary artery disease (*p* = 0.02) were identified as significant predictors of the lowest VO_2_peak (i.e., quartile 1). Left ventricular ejection fraction, septum thickness, left ventricular outflow tract gradient, dyslipidemia, smoking history, beta blocker use, and a history of peripheral vascular disease were not significant predictors of VO_2_peak (all *p* > 0.14). Figure 2 shows the multivariate predictors of VO_2_peak in HOCM patients. Multivariate analysis identified age (*p* < 0.0001), female sex (*p* = 0.001), a history of diabetes (*p* = 0.01), BMI (*p* < 0.0001), left atrial volume index (*p* = 0.04), E/e’ (*p* = 0.004), hemoglobin (*p* = 0.0004), and NT-proBNP (*p* < 0.0001) as significant independent predictors of the lowest VO_2_peak in HOCM patients.

## 4. Discussion

*Major findings:* In the present study, we determined the clinical predictors of peak exercise capacity in asymptomatic and symptomatic patients with HOCM. The novel finding of our investigation was that severely reduced exercise capacity was independently associated with female sex, a history of diabetes, age, body mass index, and echocardiography variables. Because diminished exercise capacity is predictive of mortality [9,12], the present findings highlight the critical importance of clarifying demographics and optimizing cardiovascular risk factors in HOCM patients.

*Predictors of VO_2_peak:* Patients with HCM generally exhibit reduced exercise capacity. In the present study, we found that nearly 100% of the HOCM patients had reduced exercise capacity (as indicated by VO_2_peak <80% predicted). This prevalence of HOCM patients with an abnormal VO_2_peak was substantially greater than previously reported in studies with HCM patients (i.e., 39–70%) [9,13,15,21]. As obstruction is an independent predictor of reduced exercise capacity for these patients [9,10,14,15], a likely explanation for the greater prevalence of abnormal peak exercise capacity in the present study is the diagnosed presence of obstruction in all of our patients, while previous studies have incorporated a subset of HCM with obstruction. Moreover, we found that symptoms as indicated by NYHA class were not independently predictive of diminished exercise capacity. These findings are in line with previous studies reporting that symptoms generally underestimate the severity of exercise intolerance in these patients [1,9,10,13,15,21].

In the present study, the strongest independent predictor of compromised exercise capacity in HOCM patients was female sex. Specifically, women were ~5 times more likely to have a severely reduced VO_2_peak compared to men. Furthermore, women had a lower peak O_2_pulse compared to men (10 vs. 15 mL/beat, *p* < 0.01). In agreement, most studies with HCM patients (with only a subset exhibiting obstruction) have found that female sex is predictive of reduced exercise capacity [13,14,17,21]. For example, Magri et al. found that male sex was independently associated with higher peak VO_2_ in 180 HCM patients (β = 2.84) [21]. These findings have important clinical implications, as exercise capacity is predictive of survival in HCM. In fact, a recent study in 3673 HCM patients found that female sex was associated with greater risk of mortality when analyses were adjusted for cardiovascular comorbidities, age, and symptoms [17]. Furthermore, women with HCM have more symptoms, greater left ventricular outflow tract obstruction, and an increased risk for the development of overt heart failure compared to men [12,17,25,26]. The mechanism(s) underlying the sex differences reported in exercise capacity, prognosis, and mortality in these patients are not currently known, but are likely to involve social, genetic, and/or endocrine factors [26]. For example, it has been hypothesized that HOCM is misdiagnosed in women due to a variety of factors (e.g., women being less familiar with their cardiovascular risk [27]), resulting in delayed treatment and, subsequently, more severely advanced disease when an accurate diagnosis does occur [17]. 

A history of diabetes was the second strongest predictor of compromised exercise capacity in HOCM patients. The prevalence of diabetes in the present study (~9%) was in line with previous studies of HCM patients (3–16%) [9,10,11,16,28]. In support of the relationship between diabetes and reduced exercise capacity in HOCM, diabetes is independently associated with cardiac hypertrophy (often termed “diabetic cardiomyopathy”), compromised systolic and diastolic function, and risk of heart failure [29,30]. Multiple interrelated mechanisms have been implicated in diabetic cardiomyopathy, including fibrosis, mitochondrial dysfunction, apoptosis, oxidative stress, inflammation, and microvascular dysfunction [29,31]. For example, it has been suggested that hyperglycemia-induced increases in advanced glycation end product formation impair collagen degradation, resulting in myocardial stiffness [32]. Future studies are warranted to determine the mechanisms by which diabetes contributes to exercise intolerance in HOCM patients.

Left ventricular dysfunction, as indicated by left atrial index and E/e’, was also predictive of impaired exercise capacity, with BMI and age adding additional predictive utility (OR: 0.96–1.06). These findings coincide with previous studies reporting relationships between indices of diastolic dysfunction (e.g., left atrial index, E/e’, and E/A) and impaired exercise capacity in HCM patients [33,34,35,36]. Possible underlying mechanisms responsible for the significant associations presented herein include exaggerated increases in cardiac filling pressures, pulmonary congestion, and pulmonary hypertension [37,38].

*Clinical implications:* HCM is a complex, highly heterogeneous disease in which left ventricular outflow tract obstruction worsens prognosis. CPET is important in HCM management because previous studies have suggested that symptoms are poor predictors of exercise capacity. Furthermore, CPET provides important mechanistic insight into the underlying causes of exercise intolerance, as well as providing prognostic value, in HCM. The findings of the present study are clinically relevant, and indicate the importance of clinically-determined demographic information and cardiovascular risks factors, specifically female sex and a history of diabetes, in predicting reduced exercise capacity. In addition, these findings provide specific clinical parameters that can be managed in order to ameliorate the severely impaired exercise capacity in these patients. Whether prevention of diabetes results in improved exercise tolerance and subsequently better prognosis and reduced mortality risk in HOCM patients is unknown.

*Study limitations:* The present study was retrospective and performed at a single center, and was thus susceptible to sources of bias associated with all retrospective analyses. As this study was performed in a high-volume tertiary referral center, the symptoms and exercise intolerance experienced by these HOCM patients may be more severe than in HOCM patients in the general population. It is important to note that ~15% of this large study population were asymptomatic or mildly symptomatic (i.e., NYHA I or II), which does provide these findings with additional generalizability. In addition, optimally treated HOCM patients were included in the present study. Thus, additional studies are necessary to determine if these associations exist in untreated, newly diagnosed HOCM patients. Left ventricular dimensions, right ventricular function, and pulmonary pressures were not included in the present study. Based on the significant associations between E/e’ and left atrial volume index with lowest VO_2_peak, future studies are needed to investigate the underlying mechanisms by which diastolic dysfunction impairs peak exercise capacity in HOCM patients. Lastly, the physiologic mechanisms by which female sex and a history of diabetes contribute to exercise intolerance in HOCM were not tested in the present study, and warrant future investigation.

## 5. Conclusions

This large HOCM study population demonstrated that demographic factors (i.e., female sex), echocardiography variables, and cardiovascular risk factors (i.e., BMI and a history of diabetes) predict exercise intolerance. These findings demonstrate the importance of considering clinical and echocardiographic cardiovascular risk factors when performing CPET in HOCM patients.

## Figures and Tables

**Table 1:** Characteristics of the study population.

	VO_2_peak (mL/kg/min)					
	≤14.14	14.15–17.70	17.71–21.51	≥21.52	All Patients	*p*-Value
n	294	294	295	294	1177	
Age (years)	50 ± 15	50 ± 15	54 ± 14	56 ± 14	53 ± 14	<0.001
Height (cm)	171 ± 10	173 ± 10	172 ± 10	170 ± 10	171 ± 10	0.003
Weight (kg)	94 ± 22	93 ± 20	90 ± 20	84 ± 20	91 ± 21	0.04
BMI (kg/m^2^)	22 ± 11	23 ± 10	24 ± 11	28 ± 13	24 ± 12	<0.001
FEV_1_ (% pred.) (n = 532)	74 ± 19	82 ± 18	91 ± 16	95 ± 17	85 ± 19	<0.001
Hemoglobin (g/dL)	13.3 ± 1.5	14.0 ± 1.4	14.5 ± 1.2	14.8 ± 1.3	14.1 ± 1.5	<0.001
NT-proBNP (pg/mL) *	1110 (470–2264)	714 (285–1417)	620 (236–1151)	420 (169–1092)	661 (259–1385)	<0.001
Sex						
Men	175 (60%)	196 (67%)	175 (59%)	130 (44%)	676 (57%)	<0.001
Women	119 (40%)	98 (33%)	120 (41%)	164 (56%)	501 (43%)	
NYHA Class						
I	4 (1%)	1 (0.4%)	3 (1%)	7 (3%)	15 (1%)	0.01
II	22 (8%)	40 (14%)	32 (11%)	53 (19%)	147 (13)	
III	242 (84%)	236 (84%)	242 (86%)	202 (74%)	922 (82%)	
IV	20 (7%)	5 (2%)	5 (2%)	10 (4%)	40 (4%)	
Diabetes						
No history	261 (89%)	271 (92%)	272 (92%)	269 (91%)	1073 (91%)	0.40
History	33 (11%)	23 (8%)	23 (8%)	25 (9%)	104 (9%)	
Hypertension						
No history	150 (51%)	155 (53%)	146 (50%)	144 (49%)	595 (51%)	0.80
History	144 (49%)	139 (47%)	149 (50%)	150 (51%)	582 (49%)	
Stroke						
No history	218 (95%)	225 (98%)	229 (98%)	237 (98%)	909 (97%)	0.10
History	11 (5%)	4 (2%)	4 (2%)	5 (2%)	24 (3%)	
CAD						
No history	275 (94%)	285 (97%)	280 (95%)	280 (95%)	1120 (95%)	0.20
History	19 (6%)	9 (3%)	15 (5%)	14 (5%)	57 (5%)	
Dyslipidemia						
No history	186 (63%)	178 (61%)	154 (52%)	157 (53%)	675 (57%)	0.01
History	108 (37%)	116 (39%)	141 (48%)	137 (47%)	502 (43%)	
Current Smoker						
No	179 (61%)	206 (70%)	223 (76%)	241 (82%)	849 (72%)	<0.001
Yes	115 (39%)	88 (30%)	72 (24%)	53 (18%)	328 (28%)	
Beta blocker						
No BB use	262 (89%)	266 (90%)	274 (93%)	281 (96%)	1083 (92%)	0.01
BB use	32 (11%)	28 (10%)	21 (7%)	13 (4%)	94 (8%)	
PVD						
No history	286 (97%)	289 (98%)	291 (99%)	289 (98%)	1155 (98%)	0.60
History	8 (3%)	5 (2%)	4 (1%)	5 (2%)	22 (2%)	
A-fib						
No history	250 (85%)	260 (88%)	269 (92%)	261 (89%)	1040 (88%)	0.14
History	44 (15%)	34 (12%)	26 (9%)	33 (11%)	137 (12%)	

Data presented as mean ± SD or n (%). BMI: body mass index; FEV_1_: forced expiratory volume; NYHA: New York Health Association; CAD: coronary artery disease; PVD: peripheral vascular disease; A-fib: atrial fibrillation. * Represents median and interquartile Range (IQR).

**Table 2:** Echocardiographic data stratified by peak VO_2_.

	VO_2_peak (mL/kg/min)					
	≤14.14	14.15–17.70	17.71–21.51	≥21.52	All Patients	*p*-Value
Left ventricular ejection fraction (%) (n = 1177)	66 ± 9	67 ± 8	68 ± 7	68 ± 7	67 ± 8	<0.001
Septum (mm) (n = 1177)	19 ± 6	18 ± 5	17 ± 5	18 ± 5	18 ± 5	0.03
LVOT gradient (mmHg) (n = 905)	44 ± 31	40 ± 30	39 ± 30	40 ± 31	40 ± 30	0.07
Left atrial volume index (mL/m^2^) (n = 1177)	38 ± 12	38 ± 13	38 ± 13	37 ± 17	38 ± 14	0.90
Medial E/e’ ratio (n = 1016)	20 ± 10	18 ± 8	18 ± 9	17 ± 8	18 ± 9	<0.001
Mitral E/A ratio (n = 1087)	1.4 ± 0.8	1.2 ± 0.6	1.2 ± 0.6	1.2 ± 0.6	1.3 ± 0.7	<0.001

Data presented as mean ± SD. LVOT: left ventricular outflow tract; E/e’: ratio of the peak transmitral inflow velocity (E) to the peak mitral annular velocity (e’); E/A: ratio of E to late transmitral flow velocity (A).

**Table 3:** Peak cardiopulmonary testing data stratified by peak VO_2._

	VO_2_peak (mL/kg/min)					
	≤14.14	14.15–17.70	17.71–21.51	≥21.52	All Patients	*p*-Value
Treadmill time (min)	4.8 ± 1.6	6.0 ± 1.3	7.3 ± 1.5	8.9 ± 1.5	6.7 ± 2.1	<0.001
Relative VO_2_ (mL/kg/min)	11.4 ± 2.2	16.0 ± 1.0	19.4 ± 1.1	25.4 ± 3.7	18.0 ± 5.6	<0.001
Absolute VO_2_ (L/min)	1.0 ± 0.3	1.5 ± 0.4	1.8 ± 0.4	2.2 ± 0.5	1.6 ± 0.6	<0.001
% Predicted peak VO_2_	36 ± 8	40 ± 8	44 ± 7	48 ± 7	42 ± 9	<0.001
Respiratory exchange ratio	1.12 ± 0.14	1.16 ± 0.11	1.16 ± 0.11	1.16 ± 0.10	1.15 ± 0.12	0.002
Heart rate (beats/min)	111 ± 24	123 ± 24	131 ± 24	138 ± 23	126 ± 25	<0.001
O_2_ pulse (mL/beat)	11 ± 4	13 ± 4	14 ± 4	14 ± 5	13 ± 4	<0.001
% Predicted O_2_ pulse	45 ± 10	54 ± 9	60 ± 9	70 ± 12	58 ± 13	<0.001
V_E_/VCO_2_	38 ± 10	33 ± 6	31 ± 5	31 ± 5	33 ± 7	<0.001
Breathing reserve (%) (n = 532)	54 ± 17	56 ± 15	59 ± 15	61 ± 15	57 ± 16	0.0048
Abnormal VO_2_ slope change (n = 923)	146 (70%)	124 (54%)	116 (48%)	62 (26%)	448 (49%)	<0.001
Abnormal O_2_ pulse slope change (n = 919)	140 (68%)	136 (59%)	118 (48%)	71 (30%)	465 (51%)	<0.001

Data presented as mean ± SD. VO_2_: oxygen uptake; V_E_/VCO_2_: ventilatory equivalent for carbon dioxide production.
